# Passive demultiplexed two-photon state generation from a quantum dot

**DOI:** 10.1038/s41534-025-01083-0

**Published:** 2025-08-11

**Authors:** Yusuf Karli, Iker Avila Arenas, Christian Schimpf, Ailton Jose Garcia Junior, Santanu Manna, Florian Kappe, René Schwarz, Gabriel Undeutsch, Maximilian Aigner, Melina Peter, Saimon F. Covre da Silva, Armando Rastelli, Gregor Weihs, Vikas Remesh

**Affiliations:** 1 Institut für Experimentalphysik, Universität Innsbruck, Innsbruck, Austria; 2 Cavendish Laboratory, JJ Thomson Avenue, University of Cambridge, Cambridge, UK; 3 Institute of Semiconductor and Solid State Physics, Johannes Kepler University Linz, Linz, Austria; 4 Department of Electrical Engineering, Indian Institute of Technology Delhi, Delhi, India; 5 Universidade Estadual de Campinas, Campinas, SP Brazil

**Keywords:** Quantum information, Single photons and quantum effects, Quantum dots, Quantum optics

## Abstract

High-purity multi-photon states are essential for photonic quantum computing. Among existing platforms, semiconductor quantum dots offer a promising route to scalable and deterministic multi-photon state generation. However, to fully realize their potential, we require a suitable optical excitation method. Current approaches to multi-photon generation rely on active polarization-switching elements (e.g., electro-optic modulators, EOMs) to spatio-temporally demultiplex single photons. Yet, the achievable multi-photon rate is fundamentally limited by the switching speed of the EOM. Here, we introduce a fully passive demultiplexing technique that leverages a stimulated two-photon excitation process to achieve switching rates only limited by the quantum dot lifetime. We demonstrate this method by generating two-photon states from a single quantum dot without requiring active switching elements. Our approach significantly reduces the cost of demultiplexing while shifting it to the excitation stage, enabling loss-free demultiplexing and effectively doubling the achievable multi-photon generation rate when combined with existing active demultiplexing techniques.

## Introduction

Photonic quantum computing offers a unique advantage over other quantum platforms due to the long coherence time of photons, enabling robust quantum communication, quantum information processing, and quantum simulations^1,2^. A critical requirement for these applications is the reliable generation of high-purity multi-photon states, i.e., *n* indistinguishable photons in *n* spatial modes—which serve as fundamental building blocks for quantum algorithms, error correction, quantum simulations, and advanced photonic networks^3–5^. Multi-photon states are also essential for probing quantum optical phenomena such as multi-photon interference^6,7^. The most widely used sources to produce multi-photon quantum states are the ones relying on parametric down-conversion or four-wave mixing in nonlinear crystals. However, the probabilistic nature of photon emission might pose some limitations^8^. Alternatively, epitaxially grown nanostructures such as semiconductor quantum dots (QD) are deterministic and bright sources of exceptionally pure single-photon Fock states^9–12^, holding great promise in these applications.

Several optical schemes have been developed to trigger QD sources, achieving near-unity state-preparation efficiency^13–15^, controlled polarization^16,17^, improved photon quality^18^, and robustness for practical applications^19–22^. The QD-based sources are well-compatible with various photonic integrated platforms, and can be finely tuned by controlling the growth processes^23–25^, enabling on-chip sources for advanced photonic quantum computing devices. Furthermore, via strain-tuning^26–28^, external electric or magnetic fields^29–31^, integrating into photonic cavities, one can control the spectro-temporal indistinguishability between photons emitted from multiple QDs. Recently, several research works have demonstrated interference between photons emitted from remote QDs^32,33^, albeit with heavy experimental overhead, i.e., *n* cryostats were required to conduct an *n*-photon interference experiment.

The alternate approach found in literature is to extract a single-photon stream from a *single* QD and spatio-temporally demultiplex it, typically by employing electro-optic modulators (EOMs)^34^ or reconfigurable on-chip waveguides^35^. This is the so-called *active* demultiplexing scheme. Based on this technique, several groups have successfully demonstrated multi-photon quantum interference experiments^36–40^. Typically, EOM-based demultiplexing works as follows: a resonant EOM is operated at half the photon generation rate (i.e., the laser repetition rate) to ensure that two consecutively emitted photons are routed into two orthogonal polarization states^34^. To scale it to *n* spatial modes, a total of (*n* − 1) EOMs are required. The key limitation in generating an *n*-mode photon state via a spatio-temporal demultiplexing system lies in the first and the fastest EOM—often the most inaccessible. Though high-speed fiber-based EOMs are commercially available, they introduce a few-dB of loss.

In this work, we introduce an active-element-free approach to directly trigger a two-photon state from a QD source. Our method relies on Two-Photon Excitation (TPE) of the QD followed by a precisely timed and polarization-tailored stimulation (stim) pulse-pair to achieve high-purity, two-photon emission. We have chosen TPE to excite the QD because, firstly, it is well known for producing record-high purity single photons via the biexciton ($\left\vert xx\right\rangle$)-exciton ($\left\vert x\right\rangle$)-ground state ($\left\vert g\right\rangle$) cascade^9,12,41^. Most importantly, it intrinsically enables polarization-correlated photon emission, i.e., in horizontal (H) and vertical (V) polarization states^42,43^, due to the angular momentum properties of the $\left\vert xx\right\rangle$ states. Finally, since the excitation laser energy differs from the emitted photon energy, the need for challenging cross-polarization filtering is eliminated, allowing us to double the photon rate in a given polarization. After achieving near-unity preparation of the $\left\vert xx\right\rangle$, we send the stim pulse, which is energetically tuned to the $\left\vert xx\right\rangle$-$\left\vert x\right\rangle$ transition. The polarization of the stim pulse dictates the polarization of the subsequent $\left\vert x\right\rangle$-$\left\vert g\right\rangle$ photon emission^44^. We refer to this technique as stimulated TPE (sTPE), which has recently been employed to produce high-indistinguishability photons^12,18,45^ with a tailored degree of photon number coherence^12^. It is important to note that the arrival time of the stim pulse to the TPE pulse is ~6 ps and is determined experimentally for optimal performance. In other words, exciting a single QD using sTPE pulse pairs (H and V) gives rise to a deterministic stream of high-purity single-photon pairs (H and V). Most importantly, our simplified and cost-effective approach eliminates the need for the fastest EOM in a spatio-temporal demultiplexing scheme and therefore is not switching-rate limited. This means that by tailoring the repetition rate of the laser source, one can trigger two-photon states directly from QDs, limited only by the exciton state lifetime.

## Results

### Passive demultiplexing scheme

**Fig. 1:**
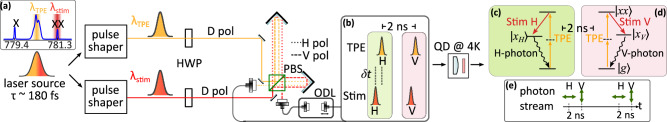
Conceptual sketch of the passive demultiplexing. Two 4*f* pulse shapers enable the preparation of the TPE (orange-shaded) and sTPE (red-shaded) pulses from a broadband femtosecond laser source. The inset **a** denotes the QD emission spectrum under TPE, where the labels *λ*_TPE_ and *λ*_stim_ denote the spectral ranges for TPE and stim pulses, respectively. The half-wave plates (HWP) ensure diagonal polarization (D pol) for both pulses before they enter a pulse-pair generator. The latter is equipped with a polarizing beamsplitter (PBS) to produce a 2 ns-time-delayed sequence of H-V polarized (H pol and V pol) TPE and stim pulses (inset **b**). To precisely set the time delay of the stim pulse from the TPE pulse, we use a fiber-optic delay line (ODL). These pulses then excite the QD kept in a cryogenic microscope at 4 K. The insets **c**, **d** represent the QD level diagrams corresponding to the H and V-polarized sTPE schemes. The inset **e** depicts the obtained H-V...H-V photon stream.

The schematic of our passive two-photon generation is described in Fig. 1. It consists of laser pulse shaping, polarization-tailored pulse-pair generation, and a cryogenic microscope hosting the QD source. In the first stage, a Ti:Sapphire laser source (Chameleon Ultra II, Coherent) is tuned to 782 nm and coupled to two independent 4*f* pulse shapers^20,22^ allowing spectral shaping of the TPE (at 780.3 nm) and stim pulses (at 781.3 nm) (Fig. 1a), whose polarizations are set to diagonal using half-wave plates (HWP) (see Fig. 1, D pol). Note that one can also generate such two-color pulses by independently tuning the wavelength and intensity via fiber-based components^46^, or a single 4*f* pulse shaper equipped with a spatial light modulator^47^. Both TPE and stim pulses enter a pulse-pair generator, where the path length differences between the two arms are adjusted to produce pulse pairs of 2 ns time delay. The choice of the 2 ns time delay is based on the reported lifetime *τ* of the QD^12,20^, such that 2 ns >> *τ*, thereby avoiding the chances of reexcitation before the state decays. Note that we use a polarizing beam splitter (PBS) in the pulse-pair generator to produce pairs of H and V-polarized TPE and stimulation pulses (Fig. 1b, green and the magenta colored boxes denoting H and V polarized pulses). The polarization of the TPE pulse is not relevant in preparing the $\left\vert xx\right\rangle$, as any linear polarization state can do so. To control the time difference *δt* between the identically polarized duo of TPE and the stim pulse, we use a fiber optic delay line (see Fig. 1, ODL). Afterwards, the TPE and stim pulses are directed to the cryostat hosting the QD sample, and the collected photons are sent to the detectors and interferometers for further analysis (see details in “Methods” section). In Fig. 1 insets (c)–(e), we depict further steps involving TPE and stim pulses, resulting in sequential H and V photon generation.

### QD excitation and control

**Fig. 2:**
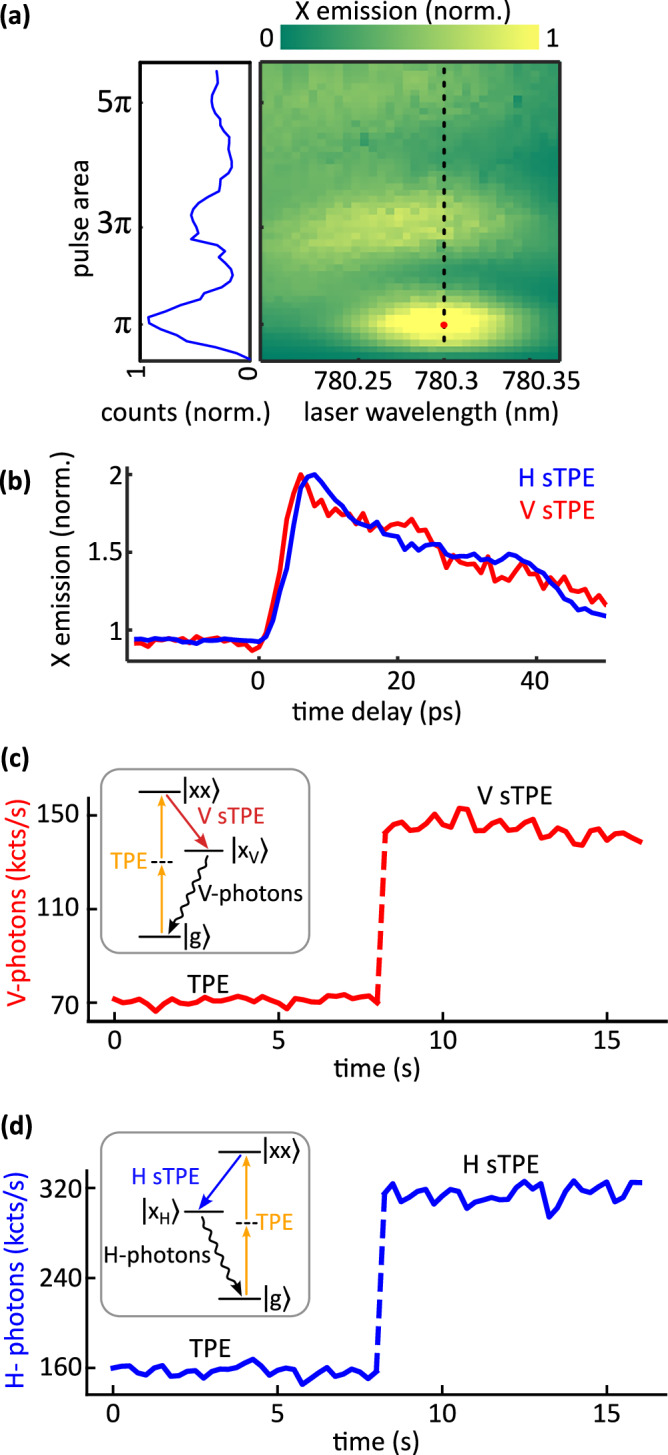
Coherent control of QD and deterministic stimulation of polarized photon states. **a** Results of the Rabi rotation experiment as a function of TPE wavelength (*x*-axis) and pulse area (*y*-axis). The dashed line indicates the TPE resonance and red dot indicates the *π* pulse condition where the QD source is operated. The left inset depicts the line cut along the dashed line. **b** Deterministic stimulation of polarized photon states via sTPE: recorded X emission as a function of the time delay between the TPE and stim pulses for H (blue curve) and V (red curve) polarizations. **c**, **d** Representative time traces of photon counts recorded under TPE (seconds 0–8) and sTPE (seconds 8–16) for both polarizations. Insets denote the respective stimulation and emission processes.

To enable TPE to $\left\vert xx\right\rangle$ we tune the laser pulse to the TPE resonance at 780.3 nm. This results in $\left\vert xx\right\rangle$ to $\left\vert x\right\rangle$ emission at 781.3 nm (hereafter called XX photon) and $\left\vert x\right\rangle$ to $\left\vert g\right\rangle$ state emission at 779.4 nm (called X photon). To verify the coherent control of $\left\vert x\right\rangle$ we perform a resonant Rabi rotation experiment by sweeping the TPE laser power and wavelength, while recording the X photon counts. The results are presented in Fig. 2a, which shows Rabi rotations in X population. Note that the observed X occupation dynamics of a QD are highly sensitive to the shape of the TPE pulse, as discussed in refs. ^20,48^.

Next, by setting both outputs of the pulse pair generator to be the TPE and sTPE *π* powers, we optimize the efficiency of sTPE. To this end, the orthogonally polarized pairs (time delay of 2 ns) of TPE pulses and stim pulses are sent to the QD, such that the stim pulse is slightly delayed by *δt* from its TPE counterpart. It is known that a precise setting of *δt* is important to ensure a near-unity stimulation of the desired polarization channel, i.e., effectively doubling the photon rate in this polarization and obtaining the highest indistinguishability of emitted X photons^12,18,45^. To verify the former, and to maximize the photon count rate under sTPE, we turn on the V-polarized stim pulse and perform a time delay scan. The results are presented in Fig. 2b. At ≈6 ps time delay, we observe that the photon counts reach twice that under TPE, clearly establishing the successful stimulation to V-polarization (red curve). Following this, we repeat the identical procedure for the H-polarized scenario and obtain nearly double the photon emission (blue curve).

Subsequently, to establish the sequential generation of H and V-polarized photon states, we proceed as follows: the first TPE pulse excites the $\left\vert xx\right\rangle$, followed by the V-polarized stim pulse deterministically triggering the V-polarized X emission. Afterwards, the second TPE pulse prepares the $\left\vert xx\right\rangle$, followed by the H-polarized stim pulse generating the corresponding X emission. This is represented in Fig. 2c, d as two exemplary 8 s time traces of photon emission at TPE and sTPE conditions for both polarizations. The H-V sequence is repeated at every 12.5 ns interval, corresponding to the original repetition rate of the laser source, resulting in an H-V H-V ... sequence of photon states. Note that the photon rate is different between H and V photon states only because of the differences in collection efficiencies of the H-collection port and V-collection ports.

### Photon characterization

To characterize the nature of the emission, we record the X emission lifetimes under sTPE (see Supplementary Information for details). The estimated lifetime for the exciton photons is found to be around 170 ps, consistent with previous reports on the GaAs QDs^12,14,19,49^.

**Fig. 3:**
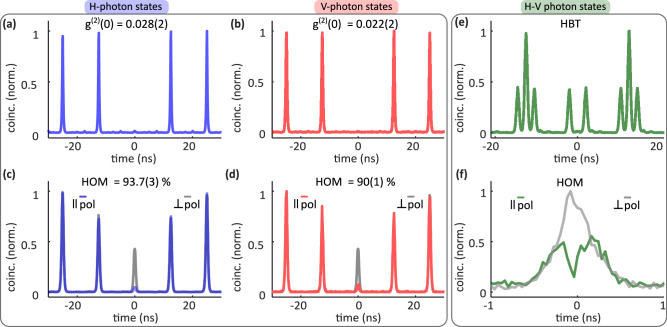
Quality of the generated two-photon states. **a**, **b** results of the ${g}^{(2)}{(0)}$ measurements, with the blue curve denoting H-polarized and the red curve denoting V-polarized photon states. **c**, **d** results of HOM coincidence measurements of the H-polarized (blue) and V-polarized photon states (red) respectively, and gray curves represent their orthogonal counterparts. **e** recorded coincidences in the HBT experiment while collecting both H and V photons. **f** the recorded coincidences in the HOM experiment for combined H-V photon states (green curve), coincidences recorded for orthogonal polarization configuration (gray curve).

To characterize the quality of the obtained two independent, orthogonally polarized single photons, we first measure the second-order correlation function *g*^(2)^(0) using Hanbury Brown and Twiss (HBT) setup, with the output ports coupled to SNSPDs (see “Methods” section). The results are presented in Fig. 3a, b. The *g*^(2)^(0) values are determined as the ratio of the mean area of the first side peaks at 12.5 ns to the central peak area, using an integration window of 1 ns for each peak. The calculated values are *g*^(2)^(0) = 0.028(2) for H-polarized photons (blue curve, panel (a)) and *g*^(2)^(0) = 0.022(2) for V-polarized photons (red curve, panel (b)), confirming the excellent single-photon properties of the generated states. The uncertainties are obtained assuming Poissonian statistics for the photon counts.

Next, we measure the single-photon indistinguishability, using the HOM setup (for details, see “Methods” and Supplementary Information). The polarization in both arms of the setup is controlled individually, and their time delay difference is set as ±12.5 ns. This enables a comparison between the cross-polarized (distinguishable) case and the co-polarized (indistinguishable) case. In Fig. 3c, d, we present the recorded coincidence histograms, where the blue and red curves denote the HOM data for H and V polarized photons in the co-polarized cases and the gray curves represent their cross-polarized counterparts. The raw HOM indistinguishability values were calculated by comparing the area under the central peak of each co-polarized case to the cross-polarized case, for 1 ns time window and Poissonian statistics for the uncertainties. The HOM visibilities are corrected using the formula^34^:

1 $$\,{\text{HOM}}=\frac{{V}_{{\rm{raw}}}+{g}^{(2)}(0)}{1-{g}^{(2)}(0)}\cdot \frac{{R}^{2}+{T}^{2}}{2RT}$$

where *R*(0.47) and *T*(0.53) represent the measured values of the reflection and transmission coefficients of the beam splitter, respectively. We measured raw HOM visibilities (*V*_raw_) of 87.6(3)% for H-polarized photons and 84.0(1)% for V-polarized photons. After correcting, the HOM visibilities increase to 93.7(3)% for H-polarized and 90(1)% for V-polarized photons, respectively. These high HOM visibilities confirm the high degree of indistinguishability of the generated single photons in both polarizations.

### Two-photon state generation

Following the photon quality measurements, we now extend our method to generate two-photon states. Initially, the HBT experiment is repeated while simultaneously recording the single-photon emission of both H and V cascades. In Fig. 3e, we present the resulting histogram, which clearly shows multiple peaks: at short time delays, we observe two peaks, while for longer delays we observe triplets, as expected for two independent single photon sources. We then look for HOM visibility between H and V-polarized photon states in a modified HOM setup where the time difference between the co- and cross-polarized arms is set to 2 ns. This corresponds to the time delay of photon emission, as decided by the pulse-pair generator. The recorded coincidences, obtained using SNSPDs and a time tagger, are shown in Fig. 3f, where the green and gray curves represent the co- and cross-polarized cases, respectively. The computed raw indistinguishability is 28(2)%.

## Discussion

**Fig. 4:**
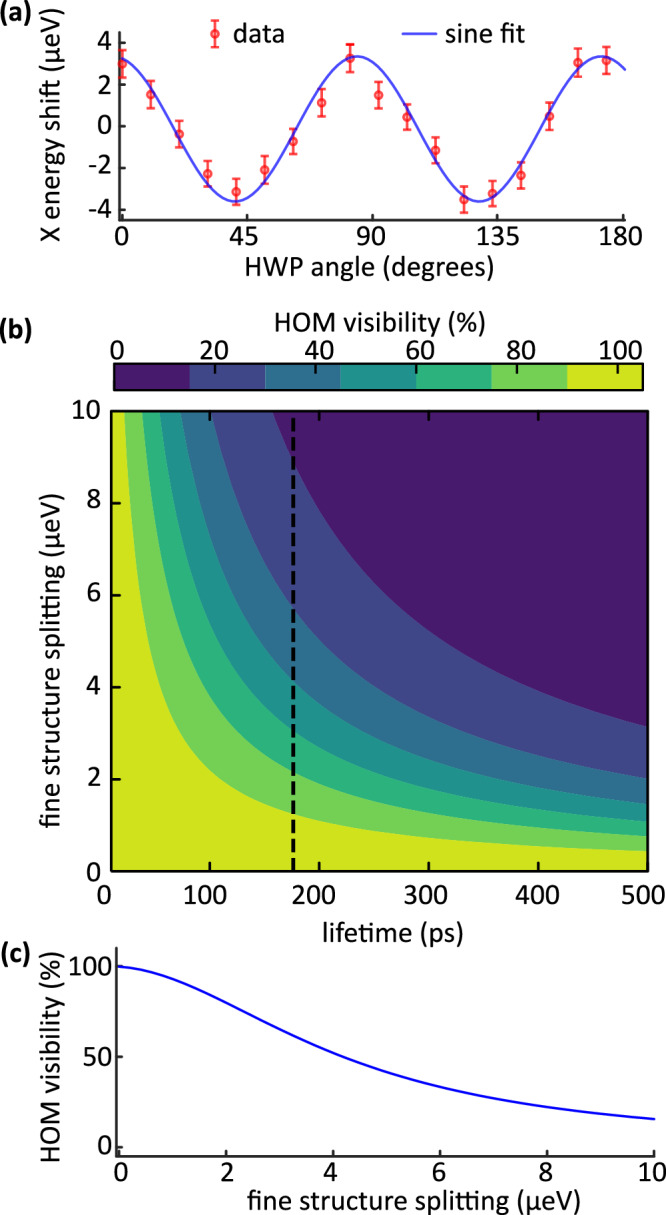
Indistinguishability limit. **a** the result of fine structure splitting measurement, **b** the simulated HOM visibility as a function of lifetime and fine structure splitting of the QD. The dashed line represents the scenario for the QD measured. **c** the line-cut of (**b**), corresponding to the lifetime of 170 ps.

To understand the limit of achievable indistinguishability in the generated 2-photon states, we start by looking into the influence of the fine structure splitting (FSS) of the QD^50^. For the investigated QD, we measured an FSS of 7.0(4) μeV, as presented in Fig. 4a. For more details on the FSS measurement, see Supplementary Information. To estimate the effect of FSS on the combined indistinguishability between H- and V-polarized photon emissions, we use the model introduced in ref. ^51^. For a difference in central frequency between H- and V-polarized photon emission of *δν* = *FSS*/*h* and for a radiative lifetime of *T*_1_, the HOM visibility V is given by

2 $$V=\frac{\,\text{Re}\,[w(z)]}{\sqrt{2\pi }\Sigma \,2{T}_{1}},\quad z=\frac{2\pi \delta \nu +i\gamma }{2\pi \sqrt{2}\Sigma },$$

with *w*(*z*) being the Faddeeva function, *Σ* the standard deviation of the inhomogeneous broadening from spectral wandering, and *γ* the dephasing. If pure dephasing like phonon broadening is neglected, *γ* = 1/*T*_1_. Figure 4b shows the behavior of *V* for *Σ* → 0 as a function of *T*_1_ and *δν*, and Fig. 4c shows the line-cut corresponding to *T*_1_ ≈ 170 ps, corresponding to the QD investigated. As our QDs are embedded in a p-i-n diode structure, spectral wandering is nearly absent. For the FSS ≈ 7 μeV, the maximum expected indistinguishability between the H- and V-polarized photons is found to be 25%, which is in agreement with the experimental value.

The indistinguishability between the H and V-polarized photon streams we generated is fundamentally limited by the FSS and lifetime of the QD. This opens up two distinct use cases for our technique. Firstly, where the QD does not provide high indistinguishability, it enables the generation of two distinct streams of single photons (H and V) from a single laser and a single QD, overcoming the need for multiple sources or multiple QDs and cryostats holding the QDs. One potential application is simultaneous secure quantum communication with two different parties without sacrificing the bit rate. Importantly, the more critical use case arises when high indistinguishability of H-V photons is available. In this case, our approach significantly enhances the existing multiplexing schemes by doubling the maximum achievable rate^36,37^. This capability could improve the performance of quantum algorithms, simulations, and error correction protocols, advantageous for quantum technologies. We identify that higher indistinguishability could be achieved in two ways: lower FSS or lower lifetimes. While the former is achievable via optimized growth processes^23–25^, strain-tuning^26–28^, applying external magnetic fields^29^ or electric fields^30,31^, the latter can be achieved by integrating QDs in engineered photonic cavities such as circular Bragg gratings^52^.

Several further interesting recent advancements also align with our approach. For instance, compact, few-element GHz laser sources are now feasible^53^, and on-chip Ti:Sapphire lasers are approaching commercialization^54–56^. A recent work^57^ demonstrates a quantum dot single-photon source reaching the loss-tolerant efficiency threshold^8^ for scalable photonic quantum computing. Banking on these, our technique could help generate high-brightness multi-photon states at GHz clock rate from a QD. In tandem, recent demonstrations of fiber-coupled single-photon sources^52,58^, and techniques for robust, plug-and-play optical excitation of QD sources based on chirped fiber Bragg gratings^19,20^ suggest that compact and field-deployable QD photon sources are forthcoming.

To summarize, we have demonstrated a fully passive demultiplexing method to generate two-photon states from a QD. Our technique relies on TPE of a QD, followed by a precisely timed, polarization-tailored stimulation pulse. Using this approach, we successfully generated a stream of H- and V-polarized photons directly from the QD. This method is conceptually similar to exciting a QD source operating at 160 MHz and then performing targeted spatio-temporal demultiplexing with active elements at 80 MHz, pushing the limits of current state-of-the-art free-space EOM technology (for further discussion, see Supplementary Information). In contrast, the switching rate of our passive multiplexing technique is limited only by the exciton state lifetime. Since the QD lifetimes allow for a GHz-rate excitation cycle, this approach removes not only the need for a fast EOM but also reduces photon loss by moving the demultiplexing step to the excitation process-thereby overcoming one of the crucial obstacles on the path to photonic quantum computing.

## Methods

### Sample and cryogenic microscopy

Our sample consists of a GaAs/AlGaAs QDs grown using molecular beam epitaxy with local droplet etching^59^, emitting around 780 nm (see details of the sample growth and the structure in Supplementary Fig. 1). The QDs are embedded into a p-i-n diode structure to control the local charge environment (see details of bias-voltage-dependent QD emission in Supplementary Fig. 2). The excitation laser source is a Ti:Sapphire laser source (Chameleon Ultra II, Coherent) producing 180 fs pulses (≈5 nm spectral intensity full width half maximum), coupled to two independent 4*f* pulse shapers, enabling spectral shaping of both the TPE and stimulating (stim) pulses (see design details in refs. ^22,48^). The intensities of the TPE and stim pulses are individually controlled via electronic variable optical attenuators (VOA, V800PA, Thorlabs) and the arrival time of the stim pulse is precisely controlled via a fiber-optic delay line (ODL-300, OZ Optics). The two beams are combined at a 10:90 beam splitter near the optical window of a closed-cycle cryostat (4 K, AttodryXS, attocube). The collected single-photon emission at exciton energy (X) from the QD is spectrally filtered using a home-built monochromator consisting of narrow-band notch filters (FWHM 0.3 nm, Optigrate) and separated into H and V components, and detected using superconducting nanowire single-photon detectors (Eos, Single Quantum) coupled to a time tagger (Time Tagger Ultra, Swabian Instruments). To determine the photon quality, we employ a HBT setup measuring ${g}^{(2)}{(0)}$ and a Hong-Ou-Mandel (HOM) setup measuring the indistinguishability of successively emitted photons. To measure the spectra, collected photons are routed to a single-photon sensitive spectrometer (with a 1200 grooves/mm grating, Acton SP-2750, Princeton Instruments) equipped with a charge-coupled device camera (iKon-M, Andor Technology).

### Single-photon purity measurement

Experimentally, we measured the single photon purity of the exciton emission from the quantum dot using an fiber beam splitter (FBS) and SNSPDs to detect the photons at each output, and the time tagger to record the coincidences. We then track the number of coincidences at these outputs for different time delays during 30 min. If our source produces single photons we expect zero coincidences at zero time delay, while for a time delay of the laser pulse repetition period, we expect maximal counts. We calculate the number of coincidences by summing the counts of each peak over 1 ns synchronized with the laser pulse.

### HOM interference measurement

The indistinguishability of sequentially emitted QD photons are measured in a HOM setup (see Supplementary Fig. 5). It consists of a half-wave plate followed by a PBS, which directs incoming photons into two separate paths of orthogonal polarizations. One of these paths includes an optical delay line (*Δt*) adjusted to the temporal separation between consecutively emitted photons. As a result, in our HOM setup, the indistinguishability between two consecutively emitted photons is measured. After the delay stage, an HWP is used to control the photon polarization, enabling the adjustment between parallel and orthogonal polarization states. The two paths are then recombined at a 50:50 fiber beam splitter, where the interference occurs. The photons are subsequently detected, and coincidence events are recorded. The degree of photon indistinguishability is determined by comparing the coincidence counts in parallel and orthogonal polarization configurations. If the photons are indistinguishable, no coincidences should be observed in the parallel configuration. In contrast, distinguishable photons do not interfere and would produce a high amount of coincidences. For measuring the HOM interference with photons that are initially horizontally (H) or vertically (V) polarized, the first HWP is set to rotate the polarization to a diagonal basis, ensuring that the PBS splits the photons evenly between the two paths. For measuring interference between photons of orthogonal polarizations (H to V), the HWP is adjusted so that photons of one polarization (the early-arriving ones) are sent through the longer (i.e., delayed) path, while the other polarization photons (i.e., the late-arriving photons) take the shorter path. Note that *Δt* needs to be adjusted differently for these two cases.

## Supplementary information

Supplementary Information

## Data Availability

The datasets generated during the current study are available in the Zenodo repository, 10.5281/zenodo.15191496.

## Code availability

The code used for the simulation during the current study is available from the corresponding authors on reasonable request.
