# Supplementary material for: Passive demultiplexed two-photon state generation from a quantum dot
Source: npj Quantum Inf. 2025 Aug 11;11(1):139. doi: 10.1038/s41534-025-01083-0 (PMC12339358; doi:10.1038/s41534-025-01083-0)
Supplement: Supplementary file 1 — Supplementary Information [file 41534_2025_1083_MOESM1_ESM.pdf]

Supplementary Information to
Passive Demultiplexed Two-photon State Generation from a Quantum Dot

Yusuf Karli,[1,2,*] Iker Avila Arenas,[1,*] Christian Schimpf,[2] Ailton Jose Garcia Junior,[3] Santanu Manna,[3,4] Florian Kappe,[1] René Schwarz,[1] Gabriel Undeutsch,[3] Maximilian Aigner,[3] Melina Peter,[3] Saimon F Covre da Silva,[3,5] Armando Rastelli,[3] Gregor Weihs,[1] and Vikas Remesh[1]

[1]*Institut für Experimentalphysik, Universität Innsbruck, 6020 Innsbruck, Austria*
[2]*Cavendish Laboratory, JJ Thomson Avenue, University of Cambridge, CB3 0HE Cambridge, UK*
[3]*Institute of Semiconductor and Solid State Physics, Johannes Kepler University Linz, 4040 Linz, Austria*
[4]*Department of Electrical Engineering, Indian Institute of Technology Delhi, Delhi 110016, India*
[5]*Universidade Estadual de Campinas, Campinas- SP, 13083-970, Brazil*
Date: Monday 7th July, 2025
E-mail: yk441@cam.ac.uk - vikas.remesh@uibk.ac.at

SUPPLEMENTARY INFORMATION

Quantum Dot sample structure

The sample was grown by Molecular Beam Epitaxy (MBE) on a semi-insulating GaAs (001) substrate. The schematic sample structure can be seen in Fig. 1 with all layer thicknesses. After in-situ deoxidation and a buffer layer of GaAs, an AlAs/GaAs superlattice (30 x ($2.5\,\mathrm{nm}/2.5\,\mathrm{nm}$)) is grown to planarize the surface and bury possible impurities present at the wafer surface. This is followed by another buffer layer. There is a distributed Bragg reflector (DBR) below the QDs. It consists of 10 pairs of $Al_{0.95}Ga_{0.05}As$ and $Al_{0.15}Ga_{0.85}As$ with an additinal $Al_{0.95}Ga_{0.05}As$ layer to match the anti-node of the electric field with the QD position in the center of the cavity, which is capped with another low reflectivity DBR (for bilayers) at the top. For the n-side of the diode $Al_{0.15}Ga_{0.85}As$ is doped with Silicon (n_{Si}=7.4$\cdot 10^{17}$ cm^{-3}). It acts as an electron reservoir for the QDs. Only 15% of Al is used for the doped layer to avoid the formation of DX centers [1]. The tunneling barrier between n-doped layer and the QDs consists of a $5\,\mathrm{nm}$ $Al_{0.15}Ga_{0.85}As$ layer deposited at relatively low substrate temperature to reduce Si segregation, followed by a higher temperature $10\,\mathrm{nm}$ $Al_{0.15}Ga_{0.85}As$ layer, as well as a $15\,\mathrm{nm}$ $Al_{0.33}Ga_{0.67}As$ layer. QDs are grown via local droplet etching, where aluminium is evaporated without arsenic background and forms droplets on the last tunneling barrier layer ($Al_{0.33}Ga_{0.67}As$). Holes are etched into the surface during exposure to As due to the As gradient between droplets and surface, resulting in about $8\,\mathrm{nm}$ deep nanoholes [2]. They are subsequently filled with GaAs to form QDs and then capped with another layer of $Al_{0.33}Ga_{0.67}As$. More details can be found in [3]. An $Al_{0.33}Ga_{0.67}As$ layer of thickness $268.4\,\mathrm{nm}$ separates the $Al_{0.15}Ga_{0.85}As$:C from the QDs. There is $67\,\mathrm{nm}$ with a $n_C = 5 \cdot 10^{18}$ cm^{-3} doping concentration (p+) and a highly doped ($n_C = 9 \cdot 10^{18}$ cm^{-3}) 13 nm layer (p++). After the top DBR the structure is capped with $4\,\mathrm{nm}$ of GaAs that protects the AlGaAs layers below from oxidizing.

The resulting structure holds the quantum dot in a pin diode, which allows for QD emission control as a function of the bias voltage. As shown in Figure 2 this results in various charge states yielding various emission lines. The neutral exciton emission is found near $779.4\,\mathrm{nm}$.

Thickness (nm)	Layer	
4	GaAs	cap
56.8	Al0.15Ga0.80As	x4 (DBR)
66.2	Al0.95Ga0.05As	
13.1	Al0.15Ga0.85As C: p++	highly p-doped layer
65.6	Al0.15Ga0.85As C: p	p-doped layer
268.4	Al0.33Ga0.67As	QDs
15.1	Al0.33Ga0.67As	tunneling barrier
10	Al0.15Ga0.85As	tunneling barrier
5	Al0.15Ga0.85As	tunneling barrier
75.7	Al0.15Ga0.85As Si: n	n-doped layer
20.2	Al0.15Ga0.85As	
97.9	Al0.15Ga0.85As	
66.2	Al0.95Ga0.05As	x10 (DBR)
56.8	Al0.15Ga0.80As	
66.2	Al0.95Ga0.05As	
50	GaAs	buffer
	30x GaAs(2.5nm)/AlAs(2.5nm)	Superlattice
12	GaAs	buffer

Supplementary Figure 1. **Schematic of the QD sample structure.**

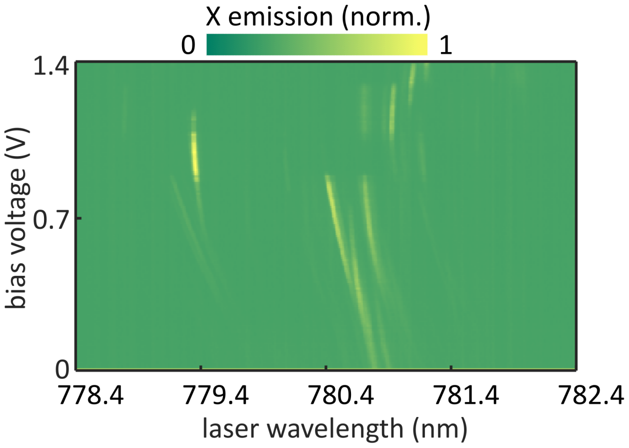

Supplementary Figure 2. **Charge control of quantum dot emission:** QD emission spectrum as a function of bias voltage across the pin diode structure, showing emission from various charge states. The neutral X emission, used as single-photon source in this manuscript, is found near 779.4 nm.

Fine Structure Splitting Measurement

The fine structure splitting (FSS) of a quantum dot can be measured via two methods: polarization-dependent spectral measurement or via lifetime analysis. The former relies on the fact that the quantum dot exciton emission shows a wavelength shift between the Horizontally polarized and the Vertically polarized excitons. The measurement of this shift is achieved by a linear polarizer (or a polarization beamsplitter in combination with a half-wave plate) at the collection path after TPE excitation, so that the exciton photon is projected onto a linear polarization before detection. To calculate the central energy, we fit a Gaussian function to the obtained spectra for each angle. In Figure 3 (a)-(f) we show the recorded spectra in the spectrometer for all the angles and the Gaussian fit for three angles of the HWP. We find that the central wavelength of the emission exhibits a small oscillation depending on the polarization. To calculate the amplitude of the oscillation, we calculate the mean central energy and plot the energy difference from this central energy for each HWP angle. The result shows that the oscillation follows a sinusoidal shape; thus, we fit a sinusoidal function to find the difference between the most extreme energies to be $7.0(4)\,\mu\text{eV}$, corresponding to the FSS value of the quantum dot.

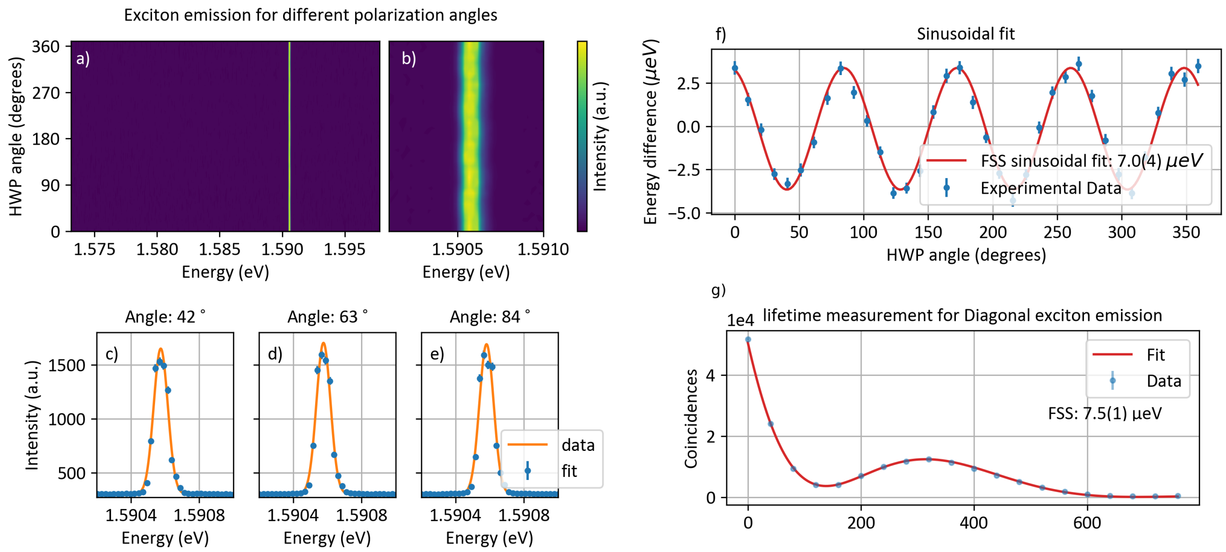

Supplementary Figure 3. Fine Structure Splitting (FSS) measurement in spectral and time domains. (a) quantum dot emission spectra showing exciton emission, measured as a function of HWP rotation angle (b) zoom-in of (a), (c)- (e) Gaussian fit to the exciton emission for three representative HWP angles, (f) exciton emission energy as a function of HWP rotation angle, computed from the data in (a), (g) FSS measurement in temporal domain, evidenced as the oscillation in exciton lifetime measurement.

An alternative way to calculate the FSS value is to measure the lifetime when the collected light is a superposition of the H and V cascades (provided one has high time-resolution detectors), in a so-called time-resolved cross-correlation. The vertically and horizontally polarized exciton photons have a time evolution given by:

$$|\psi_{x_H}(t)\rangle = e^{-\frac{t}{2\tau}} e^{-i\frac{E_H}{h}t}|H\rangle \tag{1}$$

$$|\psi_{x_V}(t)\rangle = e^{-\frac{t}{2\tau}} e^{-i\frac{E_V}{h}t}|V\rangle \tag{2}$$

where $\left|\psi_{x_p}\right\rangle$ indicate the exciton photon with polarization p, $|p\rangle$ is the polarization state in the quantum dot frame of reference, E_p is the central energy of the exciton with polarization p and τ is the exciton lifetime. An equal superposition of both cascades, like in TPE, will lead to an exciton photon state given by:

$$|\psi_x(t)\rangle = \frac{1}{\sqrt{2}}\left(|\psi_{x_V}(t)\rangle + |\psi_{x_H}(t)\rangle\right) \tag{3}$$

$$= \frac{1}{\sqrt{2}}\left(e^{-\frac{t}{2\tau}} e^{-i\frac{E_V}{h}t}|V\rangle + e^{-\frac{t}{2\tau}} e^{-i\frac{E_H}{h}t}|H\rangle\right) \tag{4}$$

$$= \frac{1}{\sqrt{2}} e^{-\frac{t}{2\tau}} e^{i\frac{E_H}{\hbar}t} \left(e^{-i\frac{S}{\hbar}t} |V\rangle + |H\rangle \right) \tag{5}$$

Where $S = E_V - E_H$ is the fine structure splitting. If the quantum dot and the laboratory frame of references are related by an angle θ, then a collection in an arbitrary diagonal polarization has a corresponding state:

$$|D\rangle = \cos(\theta)|V\rangle - \sin(\theta)|H\rangle \tag{6}$$

then the single-photon collected in this diagonal is proportional to:

$$|\langle D|\psi_x(t)\rangle\,|^2 = \frac{1}{2} e^{-t/\tau} \left| \cos(\theta) e^{-i\frac{S}{\hbar}t} - \sin(\theta) \right|^2 \tag{7}$$

$$= \frac{1}{2} e^{-t/\tau} \left(1 - 2\sin(\theta)\cos(\theta)\cos\left(\frac{St}{\hbar}\right) \right)^2 \tag{8}$$

Scaling this to N photons collected and considering an arbitrary starting time (represented as ϕ)

$$C(t) = N e^{-t/\tau} \left(1 - 2\sin(\theta)\cos(\theta)\cos\left(\frac{St}{\hbar} + \phi\right) \right)^2 \tag{9}$$

Experimentally, this can be measured by starting a clock when the biexciton photon is detected and stopping it when the exciton photon is detected. The resulting data for this is shown in Figure 3 (g), the superposition of the two cascades results in an oscillatory trend on top of an otherwise exponential decay curve. By fitting the model we find the FSS to be $7.5(1)\,\mu\text{eV}$, which agrees well with the polarization-resolved spectral analysis method.

Quantum dot lifetime measurement

The exciton state lifetimes τ are measured by various methods in our work, as described below.

In the first method, which relies on starting the clock with the TPE laser pulse and stopping the clock with the exciton photon arrival, and the computed lifetime is $175(4)\,\mathrm{ps}$. This value is represented below in Figure 4 (c). We note that the previously reported value in the supplementary information $164(1)\,\mathrm{ps}$ was an error in the fit, which we have now corrected.

In the second method, we perform lifetime measurement under sTPE, by starting a clock when the biexciton photon is detected and stopping it when the exciton photon (H or V polarization) is detected. This method provides better accuracy, as it avoids the timing uncertainty in exciton photon arrival under TPE (due to a preceding emission of the biexciton photon). One can easily observe that this timing uncertainty is removed under sTPE measurement (see Figure 4 (c), the shape of the curve carries a smoother peak compared to the case where the stim pulse is ON.) An exponential fit in the resulting decay yields the lifetimes for the horizontally (Figure 4 (a)) and vertically (Figure 4 (b)) polarized exciton states. The results found using this method are $171(1)\,\mathrm{ps}$ for the V exciton and $167(1)\,\mathrm{ps}$ for the H exciton. The mean value is $169\,\mathrm{ps}$. These are presented in Figure 4 (a) and (b). The differences in the lifetime values between H and V excitons can be attributed to the slight geometrical asymmetries in the quantum dot and uncertainties in the timing jitter of the detectors. Considering this, we have chosen the closest available data point in the simulation, $170\,\mathrm{ps}$, for the analysis of the indistinguishability between photons in each generated pair in Figure 4 in the main manuscript.

In [4], which reports measurements on an identical sample, the authors have also observed minor differences in exciton state lifetime measured under two different resonant excitation schemes. Yet, all of these computed values in both works fall in a similar range. These differences can be attributed to timing uncertainties in the detection system and differences in fitting methods.

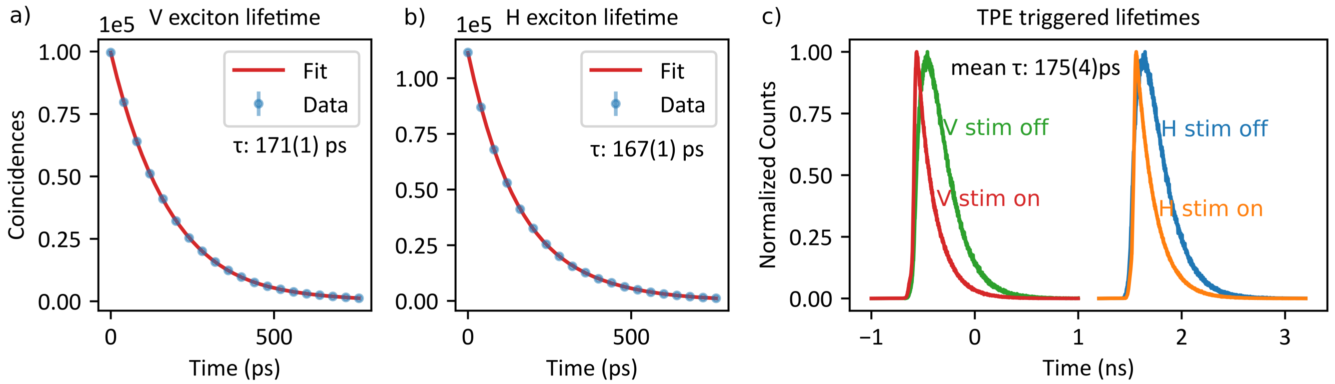

Supplementary Figure 4. **Exciton lifetime measurements:** biexciton triggered exciton lifetime measurement for Vertical (a) and Horizontal (b) photons. And TPE triggered exciton lifetime measurement with and without the stimualtion pulse (c).

HOM measurement

The setup for the HOM interference measurement is presented in Figure 5. At the entry, a half-wave plate (HWP) is followed by a polarizing beamsplitter (PBS), which directs incoming photons into two separate paths of orthogonal polarizations. One of these paths includes an optical delay line (Δt) adjusted to the temporal separation between consecutively emitted photons. As a result, in our HOM setup, the indistinguishability between two consecutively emitted photons is measured.

After the delay stage, an HWP is used to control the photon polarization, enabling the adjustment between parallel and orthogonal polarization states. The two paths are then recombined at a 50:50 fiber beam splitter (FBS), where the interference occurs. The photons are subsequently detected, and coincidence events are recorded.

The degree of photon indistinguishability is determined by comparing the coincidence counts in parallel and orthogonal polarization configurations. If the photons are indistinguishable, no coincidences should be observed in the parallel configuration. In contrast, distinguishable photons do not interfere and would produce a high amount of coincidences.

For measuring the HOM interference with photons that are initially horizontally (H) or vertically (V) polarized, the first HWP is set to rotate the polarization to a diagonal basis, ensuring that the PBS splits the photons evenly between the two paths. For measuring interference between photons of orthogonal polarizations (H to V), the HWP is adjusted so that photons of one polarization (the early-arriving ones) are sent through the longer (i.e., delayed) path, while the other polarization photons (i.e., the late-arriving photons) take the shorter path. Note that Δt needs to be adjusted differently for these two cases.

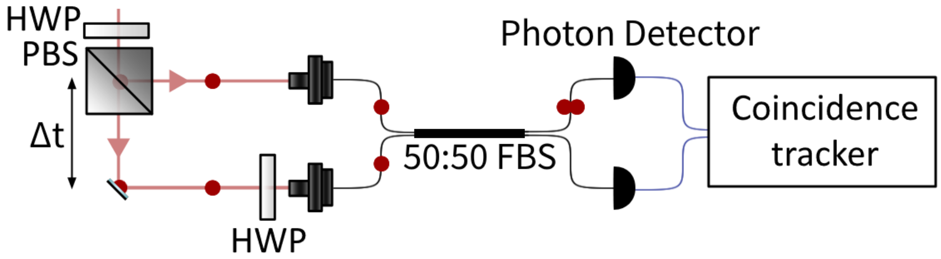

Supplementary Figure 5. **Schematic of the HOM measurement setup:** A combination of half-waveplate HWP and polarizing beamsplitter PBS is set to divert the photons into two different paths, where one path is delayed by the time difference between consecutive photons Δt. A HWP is used to adjust the polarization of the photons to be parallel or orthogonal. The interference is held in a 50:50 fiber beamsplitter FBS after which the photons are detected and the coincidences tracked.

Scaling to multi-photon state generation

For advanced quantum information processing applications, high-purity single photons are required in different spatial modes. One can either use multiple quantum dots to do this, or a single quantum dot to demultiplex the emitted photons into various spatial modes via spatio-temporal demultiplexing.

The first method of using multiple QDs to generate multi-photon states is challenging, due to the growth-induced inherent inhomogeneous broadening of QDs. This results in a spectral distribution, making every quantum dot emit at a slightly different wavelength. Triggering multiple QDs in multiple cryostats would, in general, magnify the resource overhead, particularly for a micro-photoluminescence setup involving nanopositioners, lenses, and other optical components. Note that we have demonstrated a versatile optical excitation method to trigger multiple QDs in a single cryostat, relying on chirped laser pulses [5–8]. The alternative option relies on post-growth tuning methods such as electrical/optical/magnetic/strain field control, which researchers have extensively developed.

The second approach — demultiplexing single photons from a single QD — has been successfully demonstrated in a number of studies [9–13], and even adopted by commercial quantum photonic platforms such as Quandela [14]. In typical setups, electro-optic modulators (EOMs) are used to periodically alter the polarization of photons in a time-dependent fashion. By placing a polarizing beam splitter (PBS) after the EOM, temporally adjacent photons (e.g., alternating in polarization: H-V-H-V...) can be routed into different spatial modes. An illustration is given below in Figure 6. For example, exciting a QD with an 80 MHz pulsed laser ideally produces an 80 MHz single-photon stream. Sending this stream through a 40 MHz EOM (half the laser repetition rate), with linearly polarized input (e.g., H-polarized photons), yields a polarization-alternating train: H-V-H-V..., which can be separated spatially by a PBS.

Most of the state-of-the-art works [9, 11–13] use the so-called resonant excitation of the exciton state, imposing a critical shortcoming in these approaches: the source brightness is at best 50%, enforced by the so-called cross-

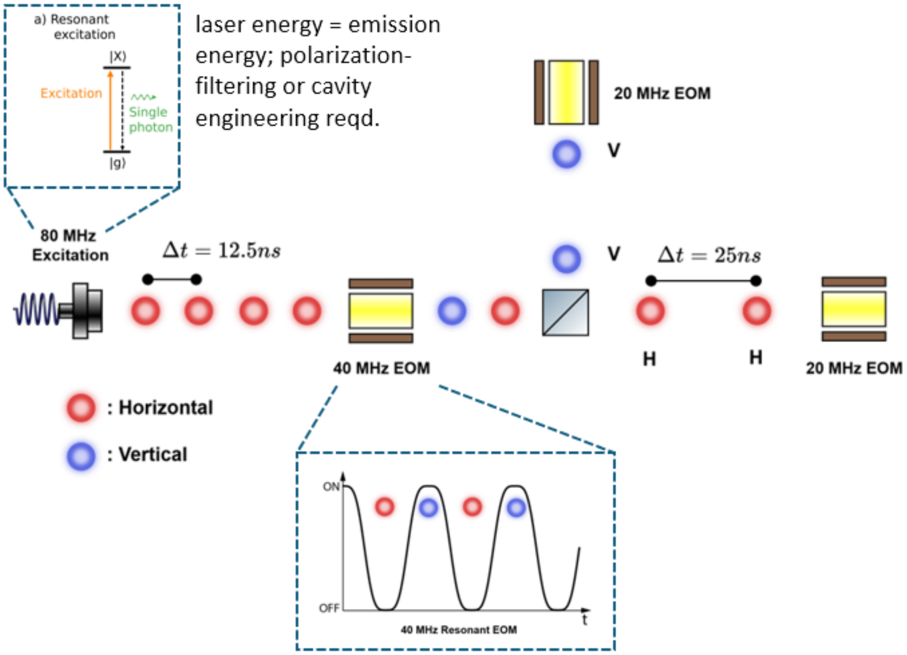

Supplementary Figure 6. **Schematic of an active demultiplexing setup.** A quantum dot is excited with an 80 MHz pulsed laser, typically resonant to the exciton state, generating a train of single photons with horizontal polarization and a temporal separation of $\Delta t = 12.5$ ns. A 40 MHz electro-optic modulator (EOM) rotates the polarization of alternating photons, resulting in a polarization sequence of H-V-H-V. A polarizing beam splitter (PBS) separates the photons into two spatial modes: horizontally (H) and vertically (V) polarized photons. Each stream, now at 40 MHz, is further directed through independent 20 MHz EOMs to achieve additional temporal demultiplexing, doubling the time separation to $\Delta t = 25$ ns.

polarization filtering [1]. Furthermore, commercially available EOMs currently support modulation rates up to 140-160 MHz, though devices beyond 100 MHz are often highly customized and costly. Even assuming a functioning 160 MHz EOM, this would limit excitation rates to 320 MHz in this scheme. However, GaAs QDs can handle much higher excitation rates due to their short lifetimes (e.g., $\approx 175 - 200$ ps without cavity enhancement), and QDs integrated with bullseye cavities have demonstrated lifetimes as short as ≈ 20 ps, allowing for excitation rates well into the GHz regime. Our work addresses these two important challenges.

Firstly, we rely on a versatile resonant and coherent optical excitation scheme called Stim. TPE. Here, the excitation laser is different from the emitted photons, and therefore, cross-polarization filtering is not required. This means that in either horizontal or vertical polarization basis, one obtains 100% photon output efficiency (ignoring the actual setup efficiency), which is the maximum amount of photons that can be obtained from an optical excitation cycle of a QD. Secondly, we eliminate the need for a first active EOM by providing a passive polarization modulation mechanism, independent of the excitation rate. This is especially advantageous at high repetition rates, where implementing fast and stable EOMs becomes technically and economically challenging. As illustrated in Figure 7, our technique can generate polarization-alternating photon trains (e.g., H-V-H-V...) passively. When this train is passed through a PBS, two spatially separated 80 MHz photon streams (all-H and all-V) are obtained. These can then be independently routed through existing demultiplexing setups using EOMs operating at lower frequencies (e.g., 40 MHz), effectively doubling the photon generation rate achievable with active schemes alone.

Our technique is therefore fully compatible with existing active demultiplexing protocols and enhances their scalability. In a QD with zero-FSS, this scheme enables photon generation in arbitrary polarization states, Scaling our technique to n photon state generation ($n \gg 2$) is highly desirable for future applications, yet, looking into current research advancements, an all-optical technique that enables this directly from a QD will require additional developments. One could perhaps envision a single, delocalized excitation that triggers multiple quantum dots and directs

[1] In [10] the authors used above-bandgap excitation scheme, which, despite being off-resonant, has limited state preparation efficiency and results in photon states with low purity. In [14], the authors employ phonon-assisted preparation, an off-resonant scheme with theoretically around 90% preparation efficiency, however, at the expense of high power and stringent spectral filtering. For resonant excitation, there are geometrically birefringent cavities [15] or waveguide strategies that spatially separate the excitation and collection light [16] to improve the brightness above 50%, however, these require extensive and specialized nanofabrication.

the emission to different spatial modes. At the same time, an approach that combines multiple quantum dots, supported by active tuning techniques such as electric field/ strain field/ temperature/ magnetic field, together with the spatio-temporal demultiplexing method, is a challenging, yet a realistic goal in the current scenario.

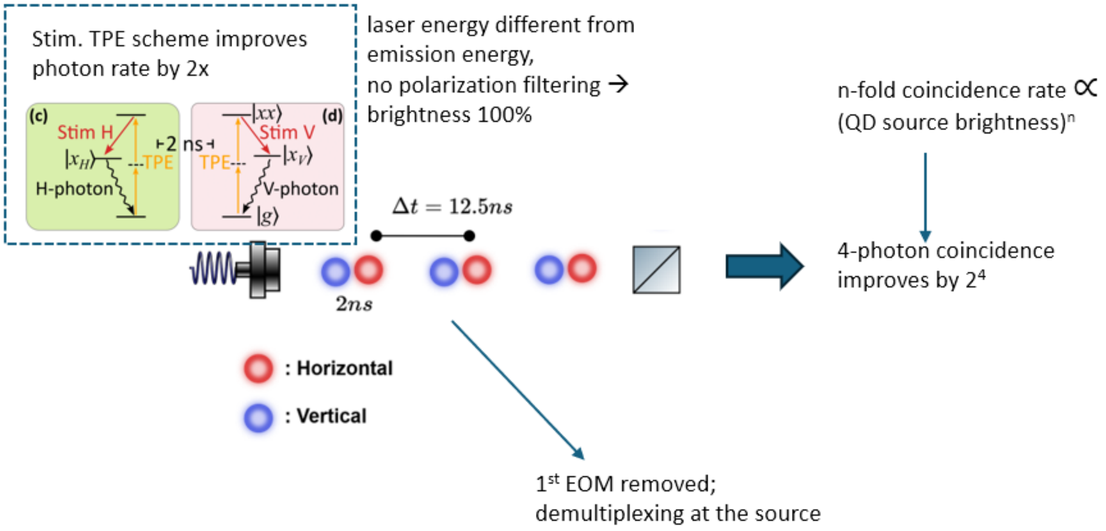

Supplementary Figure 7. **Hybrid passive-active demultiplexing scheme.** A quantum dot is excited under stimulated TPE with an orthogonally polarized pair of stimulation pulses, producing a polarization-alternating train (H-V-H-V...) with a chosen temporal separation (here $\Delta t = 2$ ns) between each H-V photons. This replaces the need for the first fast electro-optic modulator (EOM). A polarizing beam splitter (PBS) separates the stream into horizontal and vertical components, each with a photon rate of 80 MHz and a temporal spacing of $\Delta t = 12.5$ ns. These streams can be then fed into appropriate EOM-based active demultiplexers.

REFERENCES

* These authors contributed equally
[1] P. M. Mooney, Journal of Applied Physics **67**, R1–R26 (1990).
[2] C. Heyn, A. Stemmann, M. Klingbeil, C. Strelow, T. Köppen, S. Mendach, and W. Hansen, Journal of Crystal Growth **323**, 263–266 (2011).
[3] S. F. Covre da Silva, G. Undeutsch, B. Lehner, S. Manna, T. M. Krieger, M. Reindl, C. Schimpf, R. Trotta, and A. Rastelli, Appl. Phys. Lett. **119**, 120502 (2021).
[4] G. Undeutsch, M. Aigner, A. J. Garcia, J. Reindl, M. Peter, S. Mader, C. Weidinger, S. F. Covre da Silva, S. Manna, E. Schöll, and A. Rastelli, Nano Letters **25**, 7121–7127 (2025).
[5] F. Kappe, Y. Karli, T. K. Bracht, S. F. C. da Silva, T. Seidelmann, V. M. Axt, A. Rastelli, G. Weihs, D. E. Reiter, and V. Remesh, Materials for Quantum Technology **3**, 025006 (2023).
[6] F. Kappe, Y. Karli, G. Wilbur, R. G. Krämer, S. Ghosh, R. Schwarz, M. Kaiser, T. K. Bracht, D. E. Reiter, S. Nolte, *et al.*, Adv Quantum Technol , 2300352 (2024).
[7] V. Remesh, R. G. Krämer, R. Schwarz, F. Kappe, Y. Karli, M. P. Siems, T. K. Bracht, S. F. C. d. Silva, A. Rastelli, D. E. Reiter, D. Richter, S. Nolte, and G. Weihs, APL Photonics **8**, 101301 (2023), 2306.11635.
[8] Y. Karli, R. Schwarz, F. Kappe, D. A. Vajner, R. G. Krämer, T. K. Bracht, S. F. Covre da Silva, D. Richter, S. Nolte, A. Rastelli, *et al.*, Appl. Phys. Lett. **125**, 10.1063/5.0241504 (2024).
[9] F. Lenzini, B. Haylock, J. C. Loredo, R. A. Abrahao, N. A. Zakaria, S. Kasture, I. Sagnes, A. Lemaitre, H.-P. Phan, D. V. Dao, *et al.*, Laser Photonics Rev. **11**, 1600297 (2017).
[10] T. Hummel, C. Ouellet-Plamondon, E. Ugur, I. Kulkova, T. Lund-Hansen, M. A. Broome, R. Uppu, and P. Lodahl, Applied Physics Letters **115**, 10.1063/1.5096979 (2019).
[11] H. Wang, J. Qin, X. Ding, M.-C. Chen, S. Chen, X. You, Y.-M. He, X. Jiang, L. You, Z. Wang, *et al.*, Phys. Rev. Lett. **123**, 250503 (2019).
[12] L. M. Hansen, L. Carosini, L. Jehle, F. Giorgino, R. Houvenaghel, M. Vyvlecka, J. C. Loredo, and P. Walther, Optica Quantum **1**, 1 (2023).
[13] J. Münzberg, F. Draxl, S. F. Covre da Silva, Y. Karli, S. Manna, A. Rastelli, G. Weihs, and R. Keil, APL Photonics **7**, 070802 (2022).
[14] N. Maring, A. Fyrillas, M. Pont, E. Ivanov, P. Stepanov, N. Margaria, W. Hease, A. Pishchagin, A. Lemaître, I. Sagnes, *et al.*, Nat Photon **18**, 603 (2024).
[15] H. Wang, Y.-M. He, T.-H. Chung, H. Hu, Y. Yu, S. Chen, X. Ding, M.-C. Chen, J. Qin, X. Yang, R.-Z. Liu, Z.-C. Duan, J.-P. Li, S. Gerhardt, K. Winkler, J. Jurkat, L.-J. Wang, N. Gregersen, Y.-H. Huo, Q. Dai, S. Yu, S. Höfling, C.-Y. Lu, and J.-W. Pan, Nature Photonics **13**, 770–775 (2019).
[16] R. Uppu, F. T. Pedersen, Y. Wang, C. T. Olesen, C. Papon, X. Zhou, L. Midolo, S. Scholz, A. D. Wieck, A. Ludwig, and P. Lodahl, Sci. Adv. **6**, eabc8268 (2020).
